# Advances in Analysis of Biodistribution of Exosomes by Molecular Imaging

**DOI:** 10.3390/ijms21020665

**Published:** 2020-01-19

**Authors:** Yong Weon Yi, Jun Ho Lee, Sang-Yeob Kim, Chan-Gi Pack, Dae Hyun Ha, Sang Rae Park, Jinkwon Youn, Byong Seung Cho

**Affiliations:** 1 ExoCoBio Exosome Institute (EEI), ExoCoBio Inc., Seoul 08594, Korea; yongweon.yi@exocobio.com (Y.W.Y.); junho.lee@exocobio.com (J.H.L.); dh.ha@exocobio.com (D.H.H.); sangrae.park@exocobio.com (S.R.P.); jinkwon.youn@exocobio.com (J.Y.); 2 Department of Convergence Medicine, University of Ulsan College of Medicine and Asan Medical Center, Seoul 05505, Korea; sykim3yk@amc.seoul.kr (S.-Y.K.); changipack@amc.seoul.kr (C.-G.P.); 3 Asan Institute for Life Sciences, Asan Medical Center, Seoul 05505, Korea

**Keywords:** biodistribution, exosomes, labeling, molecular imaging, therapeutics

## Abstract

Exosomes are nano-sized membranous vesicles produced by nearly all types of cells. Since exosome-like vesicles are produced in an evolutionarily conserved manner for information and function transfer from the originating cells to recipient cells, an increasing number of studies have focused on their application as therapeutic agents, drug delivery vehicles, and diagnostic targets. Analysis of the in vivo distribution of exosomes is a prerequisite for the development of exosome-based therapeutics and drug delivery vehicles with accurate prediction of therapeutic dose and potential side effects. Various attempts to evaluate the biodistribution of exosomes obtained from different sources have been reported. In this review, we examined the current trends and the advantages and disadvantages of the methods used to determine the biodistribution of exosomes by molecular imaging. We also reviewed 29 publications to compare the methods employed to isolate, analyze, and label exosomes as well as to determine the biodistribution of labeled exosomes.

## 1. Introduction

**Figure 1:**
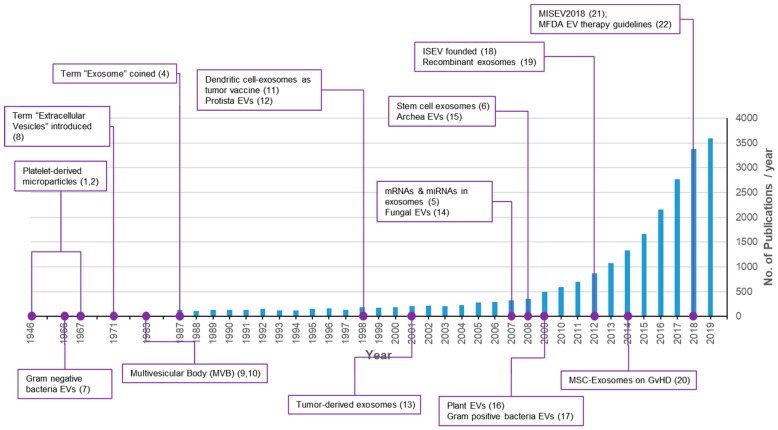
Trends of publications and major discoveries regarding exosomes. The number of publications was retrieved with a PubMed search using the keywords exosomes, exosome, extracellular vesicles, extracellular vesicle, and platelet-derived particles on 17 October 2019.

According to studies conducted over the last half century, nearly all cells on earth produce exosomes or exosome-like particles consisting of a lipid bilayer membrane [1]. The shedding of exosomes is an evolutionarily well-conserved phenomenon found in all biological kingdoms [1]. The discovery of exosomes occurred in the 1940s and platelet-derived particles in normal plasma were first reported in 1946 [2] followed by a re-description as platelet dust in 1967 [3]. However, exosomes received little attention for several decades because they were regarded as cellular garbage bins [4]. In the mid-2000s, important discoveries regarding exosomes changed this trend. In 2007, the transfer of genetic materials such as mRNAs and miRNAs in exosomes was reported [5]. Research on exosomes has been increased explosively since then, with more than 3000 papers published annually in 2018 and 2019 (Figure 1) [1,2,4,5,6,7,8,9,10,11,12,13,14,15,16,17,18,19,20,21,22]. Exosomes, ranging 100–200 nm, from stem cells were reported to mediate the paracrine therapeutic effects of stem cells [6]. Exosomes are important mediators of signal transfer in both multicellular and unicellular organisms. They are also important signaling mediators across species [14,23]. In addition to basic research, medical and healthcare industrial applications of exosomes for the development of therapeutics, drug delivery vehicles, and liquid biopsies are rapidly progressing [24,25,26].

## 2. Exosomes

### 2.1. Exosomes and Extracellular Vesicles

Extracellular vesicles (EVs) are lipid bilayered vesicles shed by cells. Three major types of EVs have been characterized according to their biogenesis: (1) exosomes are produced through the most complex process; specifically, inward budding of the cellular membrane results in the formation of early endosomes. Another inward budding of the early endosomal membrane results in multivesicular bodies (MVBs). Finally, fusion of MVBs with the plasma membrane sheds exosomes toward the extracellular space. The diameter of exosomes ranges from 30 to 200 nm; (2) microvesicles are produced from simple outward budding of the plasma membrane. The size of microvesicles are known to be from 100 or 200 to 1000 nm; and (3) apoptotic bodies are produced as a result of apoptotic cell death [27]. The apoptotic bodies are the largest type of EVs with size from 500 to 2000 nm in diameter.

Since apoptotic bodies are byproducts of cell death, numerous attempts to develop EV-based therapeutics have focused on exosomes and microvesicles. Especially, exosomes are widely accepted as next generation therapeutics due to the extensive investigation of potential applications [28,29]. As mentioned, the size ranges of exosomes and microvesicles overlap and it is difficult to differentially isolate these EVs according to their size [30,31,32]. Recently, an alternative term, small extracellular vesicles (sEVs), was proposed to refer to EVs with diameters smaller than 200 nm [21]. In this review, we refer to these smaller EVs as exosomes.

Specific markers of exosomes have been reported: ALIX and TSG101 are well-established markers of exosomes, and tetraspanins such as CD9, CD63, and CD81 are specific markers on the exosomal membrane. Additionally, exosomes contain a variety of specific proteins depending on their cells of origin [31]. Interestingly, it has been reported that exosomes derived from mesenchymal stem cells (MSCs) or HEK 293T cells do not contain class I and class II human major histocompatibility complex (MHC) proteins or co-stimulatory molecules such as CD80 and CD86. The absence of these proteins on the exosomal surface suggests no immune rejection can be expected for allogeneic therapeutics [32,33,34,35]. Exosomes derived from stem cells are actively being developed as a cell-free therapy because they recapitulate the functions of stem cells such as repair, regeneration, anti-inflation, and immune modulation without the limitations and risks of stem cells themselves [30,31,36,37]. As an example, exosomes derived from MSCs have therapeutic effects on various diseases including myocardial infarction [6,38], CCl4-induced liver injury [39], graft-versus-host disease (GvHD) [20], acute and chronic kidney injury [40], and atopic dermatitis [34,41].

The size of exosomes enables their safe systemic administration through multiple routes without the risk of embolism compared to cell-based therapy [42]. There is also a low risk of tumorigenesis since exosomes cannot replicate themselves [43]. In addition, the use of exosomes would avoid various issues related to cell therapy such as the inability to sterilize the cells, short shelf-life, and limited quality control (QC) before release [41,44]. A couple of studies have also reported that exosomes from MSCs and HEK 293T did not cause toxicity in vivo or in vitro [44,45,46,47,48]. A recent study suggested that long-term repetitive injection of exosomes does not induce toxicity [45]. Nano-sized exosomes may reach and accumulate in additional tissues beyond the tissues of therapeutic interest through systemic administration. Therefore, analysis of the biodistribution following administration through the intended route is a prerequisite for the development of exosome-based therapeutics.

### 2.2. Technologies for Isolation of Exosomes

The most important hurdle to overcome for exosome-based therapy is development of the proper technologies for large scale isolation of exosomes [49]. Exosomes from different sources have been isolated with various experimental methods such as differential ultracentrifugation (UC), density gradient ultracentrifugation (DGUC), ultrafiltration (UF), size exclusion chromatography (SEC), precipitation, and tangential flow filtration (TFF) [50,51]. According to a recent report, UC is the most widely used method to isolate exosomes from conditioned media of MSCs [24]. Commercial kits, which are mostly based on the precipitation of proteins, were the second choice for exosome isolation among the 126 papers analyzed in a recent report [24].

Among various methods, TFF has been proposed as the ideal method for industrial manufacture of exosomes [51]. Compared to other methods, which have limited compliance with good manufacturing practice (GMP), the availability of GMP-compliant TFF systems may also result in validated process control and GMP documents [50]. Methods based on UC have a risk of producing exosomes with co-precipitated contaminants and functional loss due to exosome aggregation caused by high pressure during centrifugation. The media used in DGUC may inhibit the function of exosomes [51]. Commercial kits based on protein precipitation are widely used in many academic labs. However, the additives used for precipitation (e.g., polyethylene glycol (PEG)) may inhibit the biological functions of exosomes. Although SEC has the advantage of removing proteins smaller than exosomes, a low recovery rate and the potential loss of exosome function were reported [51]. In principle, SEC cannot distinguish exosomes from non-exosomal particles with similar sizes. Recent reports revealed the functional importance of proteins associated with the surface of exosomes [52,53]. These results suggest that careful selection of the proper methods is important to isolate functional exosomes without the loss of these surface-associated proteins.

### 2.3. Quality Control of Exosomes

The QC of isolated exosomes is of importance for both reproducible research and the development of therapeutics. In an international effort to establish standards for exosome analysis, the Minimal Information for Studies of Extracellular Vesicles 2018 (MISEV 2018) was suggested through a series of publications [21,54,55]. Many studies also reported on the GMP production of exosomes for the development of therapeutics with suggested release criteria [45,50,56,57,58,59,60,61]. The worldwide market for exosome-based therapy is expected to grow from 5 million USD in 2016 to 10.0 million USD in 2021, with a compound annual growth rate (CAGR) of 14.9% [62]. In terms of regulation, fast-track approval of exosome therapeutics by regulatory authorities in Korea, Italy, and China is expected [62]. The Korea Ministry of Food and Drug Safety (MFDS) published the Guideline on Quality, Non-clinical and Clinical Assessment of Extracellular Vesicles Therapy Products in 2018 [22]. As shown in Table 1, most of the criteria in the MISEV 2018 and the MFDS Guideline are quite similar. The MFDS Guideline also includes guides for the characterization of starting materials, methods for the production, isolation, and characterization of exosomes, stability testing, the consideration of non-clinical studies, toxicological evaluation, and the considerations of clinical studies.

## 3. Analysis of Exosomes Biodistribution

### 3.1. Bioimaging Modalities

Various modalities, such as bioluminescence imaging (BLI), nuclear, fluorescence, and magnetic resonance imaging (MRI) [63,64,65], have been used for in vivo imaging (Table 2). In general, BLI is known to have the highest sensitivity and high signal-to-noise ratio while nuclear imaging has the highest penetration [63]. However, BLI with luciferase requires additional administration of substrates for luciferase and is limited by the low spatial and temporal resolution. Nuclear imaging requires hazardous radioisotopes with low spatial resolution and high cost. Fluorescence imaging with near infrared (NIR) fluorescent dyes is limited by the spatial and temporal resolution. Fluorescence imaging using fluorescent proteins (FP) has the highest spatial resolution. However, the low penetration of FP fluorescence does not allow noninvasive in vivo imaging. MRI has high penetration with high spatial and temporal resolution but is limited by low sensitivity and high cost.

### 3.2. Labeling Methods for Exosomes

For in vivo imaging, exosomes have to be labeled with probes using proper methods. Methods for labeling probes include covalent binding, genetic modification, membrane integration, encapsulation (or internalization), and metabolic labeling (Table 3).

#### 3.2.1. Covalent Binding

Covalent binding can be used to label exosomes by reacting them with probes that have functional moieties. Due to the covalent bonding, labeled probes tightly bind to exosomes with minimal dissociation. However, nonspecific exosomal proteins may also be labeled when using this method. Additionally, the labeling of exosomal surface proteins may affect their function and/or structure resulting in altered interactions of the exosomes with the target cells. It was recently reported that the modification of surface proteins altered the biodistribution of exosomes [66]. According to this report, treatment of glycosidase with exosomes resulted in a slight increase in the lung distribution of exosomes in mice compared to the distribution of untreated exosomes. However, it is necessary to further explore this finding with a large number of animals to obtain more statistically significant results since only three mice per group were used in the study. Another study performed without covalent binding suggested that labeling exosomes with lipophilic dyes also slightly changes the biodistribution of exosomes. The researchers labeled exosomes containing luciferase, with a lipophilic fluorescent dye and compared the biodistribution of the exosomes with and without the lipophilic dyes [67]. The exosomes without the lipophilic dye, accumulated in the organs in the following order: lung > liver > spleen > kidney. On the contrary, the exosomes with the lipophilic dye accumulated in the organs in the following order: liver > lung and spleen. Taken together, it is necessary to develop a method to analyze the effect of exosome surface modification.

#### 3.2.2. Surface Modification

Surface modification of exosomes can be avoided by genetic modification to load probe proteins into exosomes. To date, luciferase proteins are mostly used for genetic modification (Table 4). However, genetic modification may change the property of cells and even exosomes. Uneven loading of probe proteins is another issue that needs to be addressed [68,69].

#### 3.2.3. Membrane Integration

The most widely used labeling method for exosomes is membrane integration of lipophilic fluorescent dyes. This method is simple and easy, but carries the risk of exosome aggregation [65]. Another issue with lipophilic dyes is that they can label both lipoproteins and lipid micelles. Lipophilic dyes have been widely used to analyze the biodistribution of cells for the development of cell-based therapies. A study reported that there was no transfer of lipophilic dyes such as PKH67 or Dil from labeled to unlabeled cells in co-culture conditions [70]. These results suggest that there is a low risk of background signals resulting from the transfer of lipophilic dyes released from exosome membranes to the target tissue or cells. On the other hand, the long in vivo half-life of lipophilic dyes may cause pseudo signals after the clearance of exosomes [65]. The in vivo half-life of PKH2 and PKH26 was reported to be 12 days and more than 100 days, respectively [71]. Dialkylcarbocyanine dyes, such as DiD, Dil, DiO, and DiR, are also widely used. The in vivo half-life of DiR is known to be approximately 4 weeks [72]. Taken together, it is necessary to include a control containing lipophilic dyes alone [45]. Another potential issue with the use of lipophilic dyes is the formation of micelles in the liquid because of the lipophilic nature of the dyes [73]. When PKH26 or CM-Dil was incubated in phosphate-buffered saline (PBS) without exosomes, there were detectable levels of particles. On the contrary, in our studies, no detectable particles were observed when PKH dyes were incubated in the PBS without exosomes. In addition, no detectable changes in particle numbers were observed when PKH dyes were reacted with exosomes at the appropriate concentration (unpublished observation). Again, it is important to include a negative control that consists of the lipophilic dyes in the same buffer without exosomes. Since removal of free unlabeled dyes is a prerequisite, it is also important to process this negative control using with same removal method.

#### 3.2.4. Encapsulation

Encapsulation can be applied to label exosomes, while avoiding surface modification. However, electroporation may cause the aggregation of exosomes or structural distortion of the membrane, resulting in fused exosomes [65]. When lipophilic materials are used for encapsulation, it is difficult to exclude the possibility of sustained release of internalized probes from the exosomes. It is expected that uneven distribution of transporter proteins on the exosome membrane may cause uneven loading of probes when a transporter protein is utilized for the encapsulation of probes. The expression of a specific transporter protein is also limited by the cell types.

#### 3.2.5. Metabolic Labeling

Metabolic labeling of exosomes is achievable with the addition of specific substances during the cell culture process. After the isolation of metabolically labeled exosomes, covalent binding of the probes can be achieved with click chemistry [74]. However, the addition of extra substances during cell culture may cause changes in the characteristics of the cells or exosomes.

### 3.3. Analysis of Biodistribution of Exosomes in Literature

**Figure 2:**
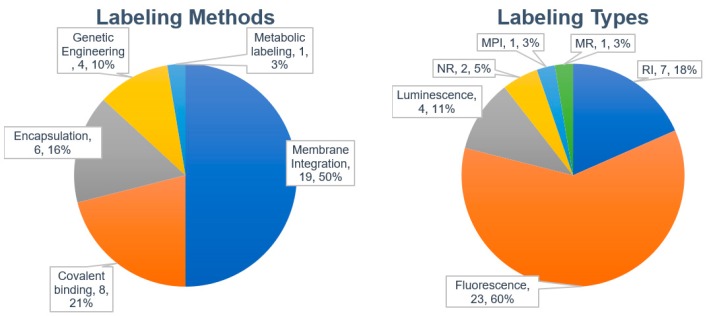
Labeling methods and probes used for labeling exosomes.

We analyzed 29 published papers that reported biodistribution studies of different exosomes or EVs (Table 4). The most widely used labeling method was membrane integration of lipophilic dyes followed by covalent binding, encapsulation (or internalization), and genetic engineering (Figure 2). Only one paper described metabolic labeling.

#### 3.3.1. Labeling Methods

All four papers involving genetic engineering described the use of luciferases. No publication was reported using fluorescent proteins (Table 4). As discussed, the low penetration of fluorescence is not suitable for noninvasive in vivo imaging (Table 2). Genetic engineering may cause changes in the characteristics of host cells or even exosomes. When genetically modified cells are used to produce labeled exosomes, the possibility of differences in the characteristics of labeled exosomes for biodistribution analysis and unlabeled exosomes for therapeutic use cannot be excluded. On the other hand, genetically labeled exosomes have an advantage in comparing their in vivo distribution with and without additional labeling. Especially, genetically labeled exosomes can be utilized to monitor the effects of surface modifications, such as covalent binding or membrane integration, which may cause structural or functional changes in the membranes of exosomes [67].

Labeling of exosomes by encapsulation has been performed with various labeling probes such as radioisotopes, nanoparticles, and fluorescent dyes (Table 4). Passive loading of probes is frequently used. An interesting example of active loading of probes is the use of transporter proteins on the membrane of exosomes. A study reported the encapsulation of glucose-coated gold nanoparticles by the GLUT1 glucose transporter on the exosomal membrane [75]. Additional transporters are expected to be available for the specific encapsulation of probes in exosomes obtained from different sources with the advancement of research. However, the distribution or abundance of transporter proteins on the exosomal membrane may cause the uneven loading of proteins. Sonication was also employed to encapsulate probes in exosomes [76]. However, sonication may cause distortion or damage to the exosomal membrane, eventually affecting the biodistribution of exosomes. Additionally, superparamagnetic iron oxide (SPIO) nanoparticles have been used to label exosomes through the transfection of exosome-producing cells [75]. It is important to recognize that the loading amount of nanoparticles is restricted by their size. The hydrodynamic radius of SPIO nanoparticles in a previous report [75] was 62 nm (https://www.magneticinsight.com/wp-content/uploads/2016/05/VivoTrax_datasheet.pdf). Since the diameter of exosomes in the study is around 100 nm [75], the loading efficiency of SPIO nanoparticles in exosomes seems to be limited.

Among the 29 papers reviewed, eight papers reported the labeling of exosomes by covalent binding to probes. The most commonly used labeling modality for covalent binding was radioisotope labeling (five out of eight) (Table 4). Fluorescent dyes (three out of eight) were also used for covalent binding. The advantage of covalent binding is the low risk of pseudo-positive signals caused by the spontaneous release of probes without covalent bonds. However, careful analysis is required since modification of surface proteins by the covalent binding of probes may change the interaction of exosomes and their target tissues or cells [73,76].

**Figure 3:**
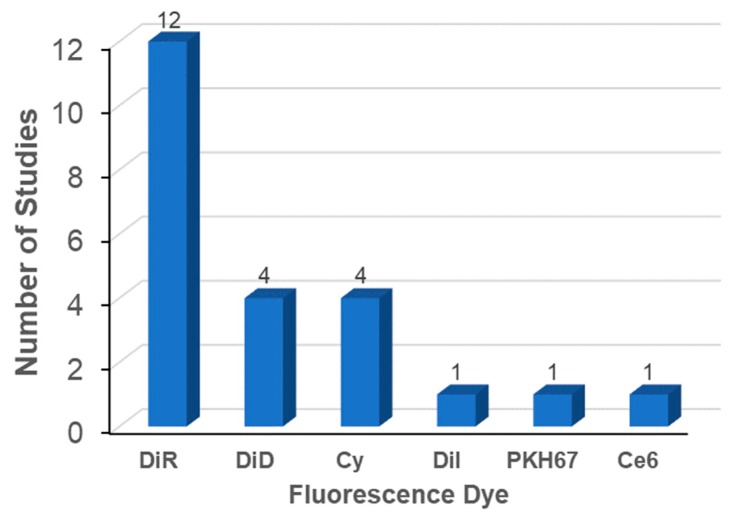
Fluorescent dyes used in biodistribution analysis of exosomes.

The most widely used labeling method is the membrane integration of lipophilic fluorescent dyes (Figure 2, left). Fifty percent of the studies evaluated used the membrane integration strategy with lipophilic fluorescent dyes (Table 4). For membrane integration, fluorescent probes were overwhelmingly selected over other methods (Figure 2, right). DiR was the most frequently used lipophilic fluorescent dye (Figure 3). DiR is a dialkylcabarbocyanine with NIR fluorescence which is ideal for in vivo imaging since it has low absorption by biological materials [98]. The FDA-approved NIR dye Indocyanine Green (ICG) is also able to label exosomes [99,100]. A potential issue is the possibility of lipophilic dyes forming micelles in the liquid [73]. Therefore, it is of utmost importance to compare the number of particles before and after labeling with lipophilic dyes. In addition, it is necessary to include a proper negative control containing the appropriate amount of lipophilic dye [45]. A buffer solution with lipophilic dyes incubated and processed using the same procedures employed for the exosomes with lipophilic dyes may also be a good negative control.

#### 3.3.2. Characterization of Exosomes

**Figure 4:**
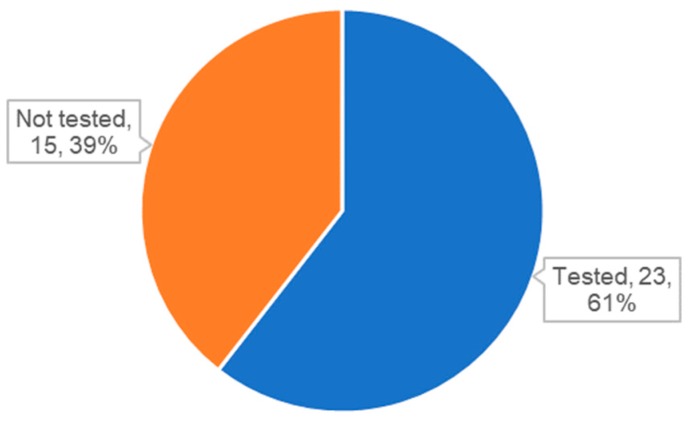
Status of analysis for specific markers for exosomes or extracellular vesicles (EVs).

One unexpected finding is that many studies used exosomes or EVs without characterization. As mentioned earlier, QC of exosomes is essential for both reproducible basic exosome research and the development of exosome therapeutics. The ISEV also proposed minimal requirements in the MISEV2018 guidelines for the identification of exosomes by analyzing specific markers [21]. Surprisingly, we found that approximately 40% of studies did not include the analysis of specific markers (Figure 4). Other than publications with exosome-like vesicles from microorganisms or exosome mimetics, 11 publications did not provide the results of specific marker analysis (Table 4). Although the results of NTA or electron microscopic analysis were reported in some cases, these results are not sufficient to confirm the identity of the exosomes used in the studies. More importantly, analysis of specific markers is especially important to compare the properties of exosomes and analyze the recovery rate between before and after labeling.

#### 3.3.3. Exosome Isolation Methods

**Figure 5:**
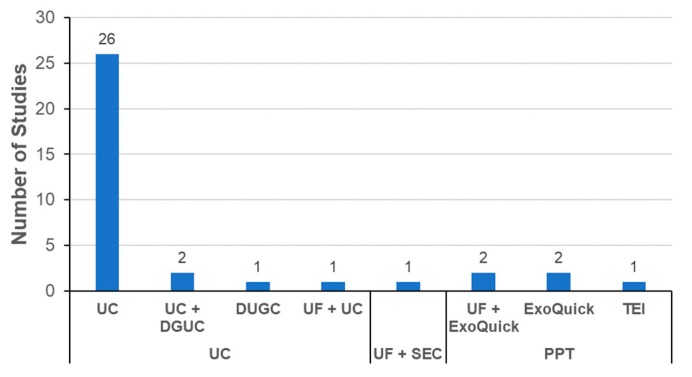
Isolation methods of exosomes in literature of exosome biodistribution.

Selection of the appropriate isolation method is essential for the industrial development of exosome-based therapeutics [50,51]. As shown in Figure 5, the dominant method for isolating exosomes is UC. This implies that UC is still the general method used to isolate exosomes in most academic settings, although the method is not ideal for the mass production of exosomes for the development of therapeutics. SEC was reported in only one publication [82]. In a few studies, precipitation with commercial kits was used to isolate exosomes. The process should be carefully monitored to determine whether the additives used for precipitation such as PEG have adverse effects on the labeling or biodistribution of exosomes. Ideally, these additives should be removed from the final exosome products before administration to an experimental animal. One publication reported that there was no significant difference in the biodistribution of exosomes isolated using UC or SEC [93].

**Figure 6:**
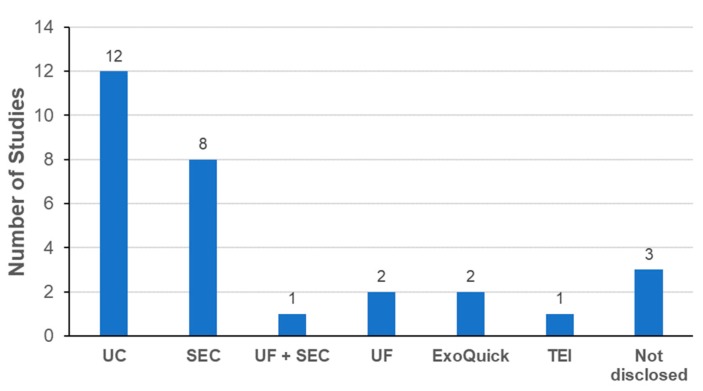
Methods to remove unlabeled probes from labeled exosomes.

Another important aspect to consider is the removal of excess unlabeled probes from labeled exosomes. UC was the most commonly used method for removing free probes in the studies evaluated (Figure 6). Interestingly, SEC was the second most frequently method for removing free probes. One drawback of SEC was the increase of sample volume with multiple fractions during the isolation process. To avoid this, methods based on the gel filtration (GF) principle are possible alternatives to conventional SEC. Commercial GF columns are already available to remove free probes by simple centrifugation without a significant increase in the sample volume [44,74,82,97]. Precipitation methods were also used to remove free probes. Again, the possibility of adverse effects from the additives used for precipitation cannot be excluded without further steps to remove the additives.

#### 3.3.4. Determination of Exosome Dose

**Figure 7:**
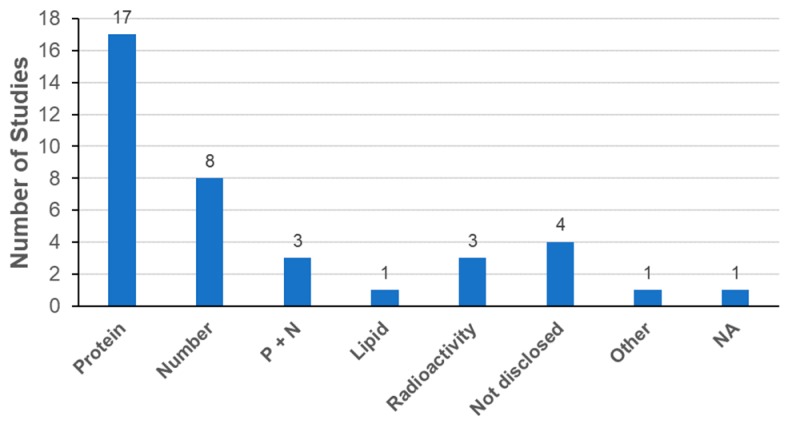
Determination of exosome dose in biodistribution. Abbreviations: Protein, total amount of proteins; number, total number of particles; P + N, total amount of proteins with total number of particles; Lipid, total amount of lipids.

Determination of the exosome dose for biodistribution analysis is another essential factor. Since exosomes are composed of lipids, proteins, and nucleic acids, it is possible to determine the exosome dose from the total amounts of lipids, proteins, or nucleic acids, respectively. It is also possible to determine the exosome dose from the total number of particles [21]. As shown in Figure 7, the most frequently used parameter for exosome dose determination was the amount of total proteins, followed by the number of particles. Parallel description of the amount of proteins and the number of particles was also reported in three publications as suggested by the ISEV in MISEV2018 [21]. The range of total proteins was from 10 to 500 μg per animal and that of the number of particles was from 2.8 × 10^9^ to approximately 3.8 × 10^11^ particles per animal (Table 4). Interestingly, all publications exclusively reported the use of mice for exosome biodistribution analysis. Recently, increasing evidence suggests that the use of zebrafish is a promising new approach to study in vivo physiology and pathology of exosomes [101]. Indeed, the transparency and small size of the zebrafish embryo enables live whole-body imaging analysis for better understanding of biodistribution including exosome uptake and fate.

#### 3.3.5. Routes of Administration

**Figure 8:**
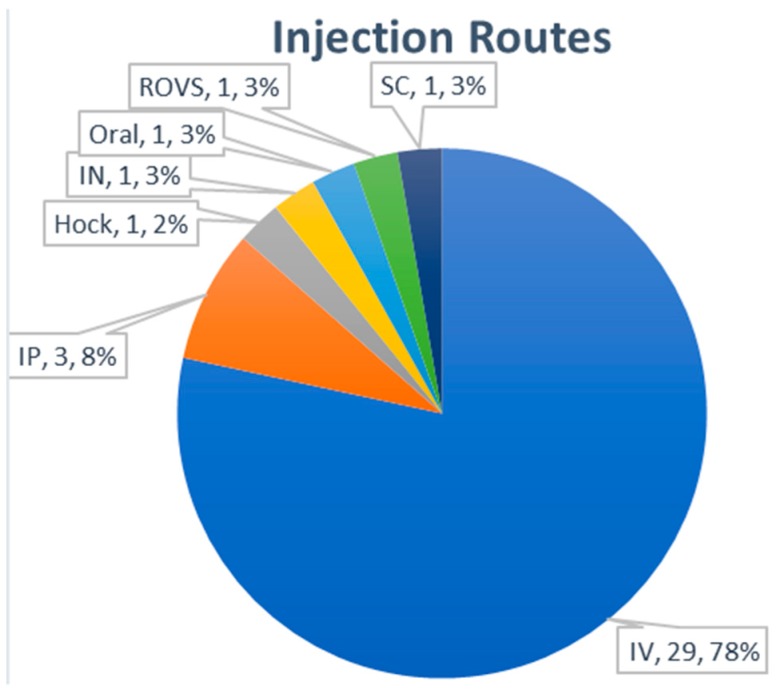
Administration route of exosomes for biodistribution analysis.

For in vivo analysis of exosome distribution, intravenous (IV) injection of exosomes was the dominant (78%) administration route (Figure 8). Three publications used intraperitoneal injection as an alternative route. The administration of exosomes through intranasal, hock, subcutaneous, and retro-orbital venous sinus routes was rarely used. The most frequent accumulation tissues for exosomes after IV injection were reported as the liver, lung, spleen, and kidney (Table 4). Although the modification of surface proteins such as glycosylation may have affected the in vivo distribution of exosomes in a few reports [66,67], additional studies with more animals seems to be necessary for more accurate analysis. It was also reported that there was a difference in the biodistribution of exosomes according to the exosome-producing cells [68]. Further studies will be needed to determine the significance of these findings.

### 3.4. Therapeutic Implication of Exosome Biodistribution

As mentioned, the information on in vivo distribution of exosomes provides basis for prediction of dose and potential side effects. In addition, it also provides the clue for target tissues of specific therapeutic application. Several studies have already provided the relevance between biodistribution and therapeutic effects.

#### 3.4.1. Natural Targeting Properties of Exosomes

Tissue tropism is dependent on the surface composition of exosomes [102]. Different integrin compositions determine the organotropism of exosomes derived from different tumors [103]. Secreted proteins such as Wnt4 and TGF-β1 have been identified to be associated with exosomes [53,104]. Wnt4-associated exosomes derived from thymic epithelial cells accumulated in the thymus of mice and this tropism was further enhanced by overexpression of Wnt4 in the originating cells, which might induce regeneration of thymus [87]. More interestingly, EVs from *Helicobactor pyroli* was reported to preferentially accumulate in stomach and induce inflammatory responses [78].

#### 3.4.2. Tumor-Homing of Exosomes

Tumor-homing exosomes could be exploited as targeting delivery vehicles. As an example, hypoxic cancer-homing exosomes, which were loaded with olaparib, demonstrated retarded tumor growth in xenograft mice [75]. Interestingly, exosomes derived from MSCs (MSC-exosomes) have been reported to exhibit tumor-homing properties similar to those of MSCs [105]. Human UC-MSC-exosomes were reported to accumulate in tumor of mouse osteosarcoma K7M2 cells in nude mice [86]. These UC-MSC-exosomes reduced proliferation of human osteosarcoma 143B and mouse osteosarcoma K7M2 cells in vitro in a dose-dependent manner by inducing apoptosis. The tumor-homing of MSC-exosomes has been successfully adopted to deliver therapeutic miRNAs to reduce tumors in xenograft mice with patient-derived pancreatic cancer [45], and syngeneic breast tumors in mice [90]. Interestingly, beyond organotropism of tumor exosomes, generalized tropism of tumor exosomes toward neoplastic tissues from different types or species have also been reported [106].

#### 3.4.3. Accumulation of MSC-Exosomes in Damaged Tissues

An interesting finding is that MSC-exosomes were preferentially accumulated in the kidneys of mice with glycerol-induced acute kidney injury compared to the distribution in normal mice [95]. The application of MSCs as a cell-based therapy for acute or chronic kidney disease has been studied [107]. MSC-exosomes have also been reported to be effective for kidney diseases in various animal models [108]. Since MSCs are known to accumulate in damaged tissues through the interactions of receptors on the MSCs and target tissues [109,110], it is highly probable that MSC-exosomes are also localized in damaged tissues due to these receptor interactions. Similarly, exosomes from endothelial progenitor cells showed accumulation in ischemic kidney to prevent ischemic injury through CXCR4–SDF-1α interaction [91].

#### 3.4.4. Tissue Targeting by Exosome Engineering

In addition to natural cell-targeting abilities, it is also possible to engineer exosomes to target specific tissues or cells [102]. PEGylation of exosomes resulted in targeted accumulation of exosomes derived from cardiosphere-derived cells in ischemic myocardium in mice [111]. Targeted delivery of exosomes by genetic modification of their surface proteins has been also been reported: (1) brain targeting by rabies viral glycoprotein (RVG) peptide or RGD motif [19,112]; and (2) tumor targeting by EGFR-specific nanobodies or HER2-specific single-chain variable fragments [113]. Recently a peptide CP05, which binds CD63, was introduced as an anchor for homing moieties to change the biodistribution of exosomes [89]. Engineered exosomes with tumor specificity could be also used to delivery chemotherapeutic agents to reduce tumors in vivo [76]. In fact, exosomes are being developed as drug carriers since they are a natural-born delivery vehicle. A wide variety of therapeutic molecules can be delivered by exosomes, including small molecules [114,115], anti-cancer drugs such as paclitaxel [116] and doxorubicin [117], and oncolytic viruses as well [116,118,119].

## 4. Conclusions

Exosomes from different cell types have unique features according to their originating cell types and are being rapidly developed as therapeutic agents, drug delivery vehicles, and liquid biopsy markers. Exosomes derived from MSCs are attractive for next generation cell-free therapeutics since they recapitulate MSC capabilities of repair/regeneration, anti-inflammation, and immune modulation and overcome the potential risk and limitations of cell-based therapeutics.

Analysis of the biodistribution of exosomes is an essential step to determine the therapeutic dose and predict the potential side effects of exosomes. However, this is extremely challenging because of the nano-range of their sizes and complex nature of their composition. QC of produced exosomes is also extremely important to ensure reproducible results. Additionally, the labeling methods and analytical modalities are limited by the characteristics of exosomes produced by living cells. A growing number of studies and advances in the methods and modalities are expected to provide proper evaluation solutions for high quality exosomes therapeutics in the near future.

## Figures and Tables

**Table 1:** Comparison of Minimal Information for Studies of Extracellular Vesicles 2018 (MISEV2018) and the Korea Ministry of Food and Drug Safety (MFDS) Guideline.

QC Criteria	MISEV2018 Recommendation	MFDS Guideline (2018)	Examples
Exosome Number (or quantification)	Global quantification by at least two methods: protein amount, particle number, lipid amount, etc.	Number of vesicles (or particles) and total protein amount or others	Nanoparticle tracking analysis (NTA) Protein quantification
Exosome Size	RPS, NTA, DLS, etc.	NTA, DLS, RPS, fluorescence correlation spectroscopy, etc.	NTA
Identity	Protein markers; Phospholipids	At least semi-quantitative method to detect proteins, RNAs, or lipids enriched in exosome	Western blot: CD9, CD63, CD81, ALIX, TSG101 FCM: CD9, CD63, CD81, and more ELISA
Purity	Ratios of two quantification figures (e.g., protein:particle) Assessment of absence of expected contamination	For proteins which are not expected to enrich in exosomes; For process impurities: serum albumin, antibiotics, etc.	ELISA for Calnexin or GM130 ELISA for impurities
Potency Assays	Dose-response assessment	Biological assay which can represent MoA	Various methods: immune-modulation, proliferation, collagen, etc.
Others	not mentioned	Mycoplasma, Sterility, Endotoxin, and Virus tests	

**Table 2:** Comparison of bioimaging modalities.

Modality	Examples	Pros	Cons
Bioluminescence Imaging [63,64]	Luciferase	Highest sensitivity (10^−15^−10^−17^ mole/L) Medium cost High signal-to-noise (compared to fluorescence)	Substrate needed Medium penetration (mm−cm) Low spatial resolution (mm) Low temporal resolution (sec−min)
Nuclear Imaging (PET/SPECT) [63,64,65]	99 mTc	Highest penetration (m) High sensitivity (10^−10^−10^−12^ mole/L) Medium temporal resolution (10 s−min)	Hazardous Low spatial resolution (mm) High cost
NIR Fluorescence Imaging [63,64]	DiR	Medium penetration (mm−cm) Medium sensitivity (10^−9^−10^−12^ mole/L) Low cost	Low spatial resolution (mm) Low temporal resolution (s−min)
Fluorescent Protein Imaging [63,64]	GFP	Highest spatial resolution (nm) Medium sensitivity	Lowest penetration (mm): does not allow noninvasive in vivo imaging
Magnetic Resonance Imaging (MRI) [63,64]	SPIO	Highest penetration (m) High spatial resolution (μm) Highest temporal resolution (min−h)	Lowest sensitivity (10^−3^−10^−5^ mole/L) High cost

**Table 3:** Comparison of labeling methods.

Labeling Methods	Pros	Cons	Reference
Covalent biding	Tight binding of probes to proteins	Cannot distinguish between exosomes vs. non-exosome proteins May change membrane protein functions which affect the interaction of exosomes with target cells	[66]
Genetic modification	Can avoid surface modification	Genetic change of cells may change the property of cells and/or exosomes Uneven loading into exosomes	[68]
Membrane integration (lipophilic fluorescent dyes)	Simple and easy	May cause clumping of exosomes Cannot distinguish between lipid proteins and micelle May cause background signals from dissociated probes May cause pseudo signals even after clearance of exosomes May affect the interaction of exosomes with target cells	[65]
Encapsulation by electroporation	May avoid surface modification	May cause aggregation or fusion of exosomes	[65]
Encapsulation by lipophilic agents	Simple and easy	May cause background signals from released probes	[65]
Transporter-dependent encapsulation	Simple and easy	Depends on transporter (e.g., GLUT1) Un-even encapsulation May cause background signals from released probes	[75]
Metabolic labeling	Covalent biding of probes by click chemistry	May change the property of cells and/or exosomes May change membrane protein functions which affect the interaction of exosomes with target cells	[74,76]

**Table 4:** Biodistribution of exosomes in literature.

Labeling Method	Modality	Nomenclature (Markers)	Cell Source	Isolation Method	Purification after Labeling	Dose (/Head)	Animal Model	Admin. Route	Imaging Method	Tissue Distribution	Ref.
Covalent binding	RI (^124I^)	EVs	MLP29 (murine liver-derived progenitor cell line)	UC (100,000 g, 70 min)	SEC (Sephadex G-25)	0.6–1.8 MBq (40–120 ng)	Mouse (BALB/cJRj)	IV hock	PET	Bladder > liver > thyroid > lung > kidney > brain	[66]
Covalent binding	Fluorescence (Cy7-NHS)	Exosomes (CD9, ALIX, TSG101)	Human U937 leukemia cells	UC (100,000 g, 2 h)	SEC (Sephadex G50)	40 μg	Mouse (BALB/c) with syngeneic CT26 colon adenocarcinoma	IV	IVIS	Liver > kidney, tumor, spleen, heart, lung, colon, brain, bladder, blood	[77]
Covalent binding	Fluorescence (Cy7-NHS)	EVs	*Helicobacter pylori*	UC (150,000 g, 3 h) and DGUC (100,000 g, 2 h)	Not disclosed	Not disclosed	Mouse (C57BL/6)	Oral	IVIS	Mouse, stomach	[78]
Covalent binding	Fluorescence (Cy7-NHS)	Bacterial EVs (OMVs)	*E. coli*	UC (150,000 g, 3 h)	UC (150,000 g, 3 h)	15 μg	Mouse (C57BL/6 and SKH1-E)	IP	IVIS	(3 h) liver > kidney > lung > spleen > small intestine (24 h) liver	[79]
Covalent binding	RI (^111^Indium)	Exosomes (CD81, CD9)	Murine B16F10 melanoma	UC (100,000 g, 90 min)	SEC (Sepharose CL-2B)	1 × 10^11^	Mouse (C57BL/6 and NSG), melanoma-bearing	IV	SPECT/CT	Liver > spleen > bone, kidney, lung	[80]
Covalent binding	RI (^131^I)	Exosomes (CD9, CD63)	Mouse MDSCs and EPCs, HEK293	UF (100 kDa) and UC (100,000 g, 70 min)	UF (100 kDa)	350 ± 50 μCi	Mouse (BALB/c or C57BL/J6) Xenograft bearing 4T1 or AT3	IV	SPECT/CT	(Tumor exosomes) tumor > liver > lung, spleen, kidney, brain, heart (MDSC-exosome) liver, lung, tumor > kidney, spleen, brain, heart (EPC-exosomes) tumor > liver > lung, kidney, brain, spleen, heart	[81]
Covalent binding	RI [^99m^Tc(CO)_3_(−H_2_O)_3_]^+^	EVs	Erythrocyte	UC (130,000 g, 30 min) and SEC	SEC (Desalting Column)	15 ± 2 Mbq	Mouse (BALB/c)	IV	SPECT/CT	Liver, bladder, spleen > kidney > lung, heart, bone	[82]
Metabolic labeling	Fluorescence (Cy3 or Cy5.5)	Exosomes (CD63)	Human MDA-MD-231 and MCF7 breast cancer cells	ExoQuick	Gel filtration (G-25)	10 μg	Mouse, athymic MDA-MB-231 or MCF7 tumor bearing	IV	IVIS	(MCF7 exosomes) liver > large and small intestines > kidney, tumor, spleen, lung, muscle, blood (MDA-MD-231 exosomes) liver > large and small intestines > lung > tumor, spleen, kidney > muscle, blood	[74]
Genetic Engineering	Luminescence (CD63-NanoLuc)	Exosomes (CD63)	HT29/CD63Nluc and HCD116/CD63Nluc	UC (110,000 g, 70 min)	NA	NA	Female mouse (Balb/c-nu/nu)	NA (SC implant of cells)	BLI (IVIS)	Stomach, intestine	[83]
Genetic Engineering	Luminescence (Renilla Luciferase; Rluc)	EVs (CD63, Alix)	CAL-62 thyroid cancer cell and MDA-MB-231 breast cancer cells	UC (100,000 g, 60 min)	NA	25 μg	Mouse (BALB/c, female) N = 3	IV	BLI (IVIS)	62/Rluc: lung> liver > spleen > kidney 62/Rluc/DiR: liver > lung, spleen 231Rluc: lung, liver > spleen > kidney	[67]
Genetic Engineering	Luminescence (Gaussia Luciferase)	EVs (CD63, ALIX)	HEK293T cells	UC (100,000 g, 90 min)	NA	100 μg	Mouse (athymic nude)	IV	BLI	Spleen, liver > lung, kidney, brain, heart, muscle	[84]
Genetic Engineering	Luminescence (Gaussia Luciferase)	Exosomes	B16-Bl6 murine melanoma cells	UC (100,000 g, 1 h)	NA	1 × 10^10^ RLU (5 μg)	Mouse (BALB/c)	IV	BLI (LAS3000)	Lung > spleen > kidney, liver, heart, brain, intestine	[85]
Membrane integration	MR (gadolinium)	Exosomes (CD9, CD63, CD81)	Human UC-MSCs	UC (120,000 g, 90 min)	UF (10 kDa)	0.015 mmol/kg	Mouse, K7M2 (human osteosarcoma) xenograft (NU/NU)	IV	MRI	Liver, spleen > tumor > lung, kidney, heart, brain	[86]
Membrane integration	Fluorescence (DiR)	Exosomes (CD9, CD63, CD81)	Human UC-MSCs	UC 120,000 g, 90 min)	Not disclosed	5 mg/kg	Mouse, K7M2 (human osteosarcoma) xenograft (NU/NU)	IV	LI-COR	Spleen > liver > tumor, lung > kidney, brain, heart	[86]
Membrane integration	Fluorescence (Dil)	Wnt4-exosomes	Mouse TEP1 (primary thymic epithelial cell)	TEI (Invitrogen)	TEI (pre-labeling)	Not disclosed	Mouse (BALB/c)	IV	IVIS	Thymus > lung, liver, spleen	[87]
Membrane integration	Fluorescence (DiR)	CVs (by sonication)	hCMEC/D3 B16	UC (60,000 rpm, 24 h)	SEC	200 μg of lipid	Mouse (FVB albino)	ROVS	IVIS	Liver > spleen, lung > brain	[88]
Membrane integration	Fluorescence (DiR)	Exosomes (ALIX, CD63, CD81, CD9, TSG101)	C2C12 murine myoblast cell	UC (100,000 g, 1 h)	Not disclosed	30 μg	Mouse (C57BL/6)	IV	IVIS	Liver > spleen > lung	[89]
Membrane integration	Fluorescence (DiR)	Exosomes	BM-MSC	UC (100,000 g, 3 h)	UC (100,000 g, ND)	8 × 10^9^	Mouse (C57BL/6) Tumor vs. non tumor	IP	IVIS	Liver, spleen, pancreas	[45]
Membrane integration	Fluorescence (PKH67)	Exosomes (CD63)	Mouse BM-MSC	UF + ExoQuick	ExoQuick	30 μg	Mouse (BALB/c) TUBO tumor	IV	IVIS	(24 h) Tumor > spleen > kidney, liver, lung	[90]
Membrane integration	Fluorescence (DiR)	Exosomes (TSG101, CD81)	Endothelial colony forming cell (ECFC)	UC (100,000 g, 90 min)	UC (100,000 g)	20 μg	Male FVB mice	IV	IVIS	(4 h) kidney > liver, heart, spleen, lung	[91]
Membrane integration	Fluorescence (DiD)	Exosomes (TSG101, CD9, HSP70; GM130-)	Murine EO771 BC cells	Combination of UF (100 kDa) and SEC	UC (100,000 g, 90 min)	20 μg (1.6 × 10^11^)	Mouse (C57BL/6 or BALB/C)	IV	IVIS organ imaging	Lung, > liver > spleen, kidney > heart > bone marrow	[92]
Membrane integration	Fluorescence (DiD)	Exosomes (TSG101, CD9, HSP70; GM130-)	Murine 4T1 BC cells	UC (100,000× *g*, 90 min)	UC (100,000 g, 90 min)	20 μg (1.2 × 10^11^)	Mouse (C57BL/6 or BALB/C)	IV	IVIS organ imaging	Lung > liver > kidney > spleen, heart, bone marrow	[92]
Membrane integration	Fluorescence (DiD)	Exosomes	Murine 67NR BC cells	UC (100,000× *g*, 90 min)	UC (100,000 g, 90 min)	20 μg (1.2 × 10^11^)	Mouse (C57BL/6 or BALB/C)	IV	IVIS organ imaging	Lung > liver > kidney > spleen, heart, bone marrow	[92]
Membrane integration	Fluorescence (DiR)	EVs	Undisclosed	NA	UC (120,000 g, 70 min) vs. UF (100 kDa)–SEC (S-400)	Undisclosed	Mouse (BALB/c)	IV	IVIS organ imaging	(UC) liver > lung, spleen > kidney (UF-SEC) liver > spleen > lung > kidney	[93]
Membrane integration	Fluorescence (DiR)	EVs (ALIX, TSG101)	HEK293T cells	UC (110,000 g, 70 min)	NA (pre-labeling before UC)	1.5 × 10^10^, 1,0 × 10^10^, 0.25 × 10^10^ p/g BW	Mouse (NMRI or C57BL/6)	IV IP SC	IVIS	(IV) liver > GI-tract, spleen > lung > pancreas (IP) liver, GI-tract, pancreas > spleen, lung (SC) GI-tract > liver > pancreas, lung > spleen	[68]
Membrane integration	Fluorescence (DiR)	EVs (ALIX, TSG101)	DC cells	UC (110,000 g, 70 min)	NA (pre-labeling before UC)	1.0 × 10^10^ p/g BW	Mouse (NMRI or C57BL/6)	IV	IVIS	Liver > spleen > GI-tract, lung > pancreas	[68]
Membrane integration	Fluorescence (DiR)	EVs (ALIX, TSG101)	C2C12 cells	UC (110,000 g, 70 min)	NA (pre-labeling before UC)	1.0 × 10^10^ p/g BW	Mouse (NMRI or C57BL/6)	IV	IVIS	Liver > spleen > GI-tract > lung > pancreas	[68]
Membrane integration	Fluorescence (DiR)	EVs (ALIX, TSG101)	B16F10 cells	UC (110,000 g, 70 min)	NA (pre-labeling before UC)	1.0 × 10^10^ p/g BW	Mouse (NMRI or C57BL/6)	IV	IVIS	Liver > GI-tract, spleen, lungs > pancreas	[68]
Membrane integration	Fluorescence (DiR)	Exosome (CD63, flotillin-1)	BMSCs	UF (3 kDa)-ExoQuick-TC	ExoQuick-TC	500 μg	C57BL/KaLwRij	IV	Fluobean 800	BM, spleen, liver	[94]
Membrane integration	Fluorescence (DiD)	EVs (CD44, CD105, CD90, α5-integrin)	MSCs	UC (100,000 g, 1 h)	UC	200 μg	Mouse CD1 with or without glycerol-induced AKI	IV	IVIS organ imaging	(24 h) liver > spleen > lung	[95]
Encapsulation	RI ^99m^Tc	Exosome mimetics	Rat RBCs	UC (100,000 g, 1 h) + DGUC	Centrifugation (not disclosed in detail)	37 Mbq	Mouse (C57BL/6, male)	IV	Gamma camera imaging	Liver, spleen, kidney > thyroid, stomach, lung, blood, intestine > heart, muscle, bone	[65]
Encapsulation	MR gold nanoparticles	Exosomes (CD9)	Human MSCs	UC (100,000 g, 70 min)	UC (100,000 g, 2 h)	2.8 × 10^9^	Mouse (C57bl/6, male)	IV IN	CT	(IV) lung, liver > spleen > kidney, brain, blood (IN) lung > spleen > kidney, brain, blood, liver	[96]
Encapsulation	RI ^99m^Tc-HMPAO	Exosome mimetic	RAW264.7	DGUC (100,000 g, 2 h)	SEC (MW3000)	7.4–14.8 Mbq (29–64 μg)	Mouse (BALB/c)	IV	SPECT/CT	(5 h) liver > kidney > spleen > intestine > lung, heart, stomach, heart > bone, muscle, blood	[97]
Encapsulation by transfection	MR SPIO	Exosomes (CD9, CD63)	MDA-MB-231	ExoQuick	NA	100 μg	Mouse	IV	MPI CT	Liver	[75]
Encapsulation by Sonication	Fluorescence Chlorin e6 (Ce6)	Tumor targeting EVs	RAW264.7	UC (100,000 g, 70 min)	UC (100,000 g, 70 min)	10 mg/kg	Mouse (BALB/c nu/nu) with HCT116 tumor	IV	Image Station 4000 MM	Tumor > liver > lung, kidney, spleen, brain, heart	[76]

**Abbreviations**: AKI, acute kidney injury; BC, breast cancer; BLI, bioluminescence imaging; BMSC, bone marrow stromal cell; BW, body weight; CV, cellular vesicle; CT, computed tomography; DGUC, density-gradient ultracentrifugation; EPCs, endothelial progenitor cells; FI, fluorescence intensity; FP, fluorescence protein; GNP, gold nanoparticle; ICP-MS, inductively coupled plasma mass spectroscopy; IN, intranasal; IV, intravenous; IP, intraperitoneal; MDSCs: myeloid derived suppressor cells; MPI, magnetic particle imaging; MR, magnetic resonance; MRI, magnetic resonance imaging; MSC, mesenchymal stem/stromal cell; NA, not applicable; ND, not determined; NR, nuclear imaging; OMV, outer membrane vesicle; RI, radioisotope; RLU, relative luminescence unit; ROVS, retro-orbital venous sinus; SC, subcutaneous; SEC, size exclusion chromatography; SPECT, single-photon emission computed tomography; SPIO, superparamagnetic iron oxide; TEI, total exosome isolation reagent; UC, ultracentrifugation; UC-MSC, umbilical cord MSC; UF, ultrafiltration.

## Abbreviations

AKI	acute kidney injury
BC	Breast cancer
BLI	bioluminescence imaging
BMSC	bone marrow stromal cell
BW	body weight
CAGR	compound annual growth rate
CV	cellular vesicle
CT	computed tomography
DGUC	density-gradient ultracentrifugation
DLS	dynamic light scattering
EPCs	endothelial progenitor cells
EVs	extracellular vesicles
FI	fluorescence intensity
FP	fluorescence protein
GMP	good manufacturing practice
GNP	gold nanoparticle
GvHD	Graft-versus-host disease
ICP-MS	inductively coupled plasma mass spectroscopy
IN	intranasal
ISEV	International Society for Extracellular Vesicles
IV	intravenous
IP	intraperitoneal
MDSCs	myeloid derived suppressor cells
MFDS	Ministry of Food and Drug Safety, Korea
MISEV	Minimal Information for Studies of Extracellular Vesicles
MHC	Major histocompatibility complex
MPI	magnetic particle imaging
MRI	magnetic resonance imaging
MSCs	mesenchymal stem/stromal cells
MVBs	multivesicular bodies
NA	not applicable
ND	not determined
NIR	near infrared
NR	nuclear imaging
NTA	nanoparticle tracking analysis
OMV	outer membrane vesicle
PEG	polyethylene glycol
PET	position-emission tomography
QCs	quality controls
RI	radioisotope
RLU	relative luminescence unit
ROVS	retro-orbital venous sinus
RPS	resistive pulse sensing
SC	subcutaneous
SEC	size exclusion chromatography
SPECT	single-photon emission computed tomography
SPIO	superparamagnetic iron oxide
TEI	total exosome isolation reagent
TFF	tangential flow filtration
UC	ultracentrifugation
UC-MSC	umbilical cord MSC
UF	ultrafiltration
